# Obstetrics

**Published:** 1907-02

**Authors:** E. S. McKee

**Affiliations:** Cincinnati. Lecturer on Clinical Gynecology, Medical College of Ohio—University of Cincinnati


### PROGRESS IN MEDICAL SCIENCE.

### Obstetrics.

By E. S. McKEE, M. D., Cincinnati.
Lecturer on Clinical Gynecology, Medical College of Ohio—University of Cincinnati.

DEATH DURING PREGNANCY; UNDETECTED CANCER OF KIDNEY.

GAULTIER (*Ann. de Gyn. et d'Obstet.*) reports that a needlewoman aged 35, in the seventh month of her third pregnancy, gained admission into a hospital on account of distressing fits of coughing which had lasted for two or three months. The symptoms simulated whooping cough. The sputum was tenacious and frothy, sometimes streaked with blood, and at others contained gelatinous masses of blood pigment. Tuberculous disease of the bronchial glands was diagnosticated. The urine was absolutely normal, and Gaultier notes that the patient never said a word about lumbar pains, so frequent in pregnant women. There was no abdominal tumor. The patient died during a very violent fit of coughing. The right lung and pleura were infected with cancerous deposit; the tracheo-bronchial glands were infected, the largest compressing the right bronchus and pneumogastric. The primary seat of cancer was found to be the right kidney, enlarged to the size of a hen's egg, the pelvis and renal vein being invaded. The fetus showed high congestion of the kidney and suprarenal capsule, with numerous hemorrhagic foci.

PAINLESS LABOR.

LAMPHEAR (*Medical Record*) gives the following description of his method: When labor has progressed to the stage in which the os uteri is well dilated and the pains are becoming distressingly severe, a hypodermic injection of one quarter of a grain of morphine and one hundreth of a grain of hydrobromate of hyoscine may be given; in one hour the forceps may be applied and labor completed without any pain whatever, even though the perineum be lacerated and sewed up. There need be no hurry; the perfect analgesia will last for hours. If the patient is not asleep at the end of an hour after the injection, a few drops of chloroform may be given by inhalation, a dram at most usually putting the patient to sleep of some hours' duration. There will be none of the nausea of a prolonged ether or chloroform narcosis, no increased danger of hemorrhage as after chloroform, and no necessity for a skilled assistant to give the anesthetic.

CRIMINAL ABORTION AND ILLEGITIMACY.

CARSTENS (*Medical Record*) finds home education and the development of self control in the young to be the remedy for illegitimacy. He says that if mothers would explain to their daughters the physiological process of reproduction there would be very little illegitimacy. As to the crime of abortion he thinks religious teaching very weak, and this failing we should impress upon the mind the great immediate danger and the chronic invalidism that follows in many cases. We should also help the unfortunate unmarried by showing them how to legitimately avoid exposure. There is, however, a deep seated reason, and that is, he thinks, the tendency to late marriage and to the correction of this as well as to home training, must we look for the cure of the evils of illegitimacy and abortion.

---

ECTOPIC GESTATION OCCURRING TWICE IN THE SAME PATIENT.

ROWSE (*Lancet*) reports a case in which, in both instances, the uterus was retroverted. The patient was a II-para in which the usual symptoms appeared toward the end of the second month. Abdominal incision showed rupture of the right tube with a pint of blood between the two layers of the broad ligament. This was evacuated by the extraperitoneal method. The ovary and tube were not removed. A similar experience happened nine years later, no intervening pregnancy having occurred. At this time behind and to the left of the uterus was a large adventitious sac containing the ovum and about a quart of blood. The clot and ovum were removed with the tube and ovary on that side. Patient recovered.

---

DOUBLE OVARIOTOMY—PREVENTION OF BAD RESULTS.

ZACHARIAS, (*Centrallblatt fuer Gynkologie*) when both ovaries are removed for benign cysts or tumors or inflammatory conditions advises that a piece of the cortex of the less involved ovary be separated from the growth removed and left in connection with the hilus. This piece should be about one millimeter thick and twenty-five to fifty millimeters in diameter, should be drawn together with fine catgut into a mass or cylinder attached to the broad ligament. Even this small portion of ovarian substance is sufficient to prevent the bad psychical and physical effects which so often follow complete castration.

---

SUDDEN DEATH AFTER LABOR.

McDANIEL (*Medical World*) reports a case of mysterious death after labor. The etiology was obscure. Meigs originated the theory that a partial detachment of the placenta during labor with weakness of the heart leads to the formation of large soft clots in the uterine sinuses, which may become detached by a subsequent powerful uterine contraction and emboli lodge in the heart and pulmonary artery. The entrance of air in the circulation through the uterine sinuses has not received recent confirmation as a cause of sudden death. Shock, it seems, may be the cause of death after labor as after any surgical operation, but its occurrence is doubted by Williams. Lusk, about twenty years ago (*Journal A. M. A.*) wrote a fine article on this subject and collated reported cases. Rupture of the genital tract may give rise to slight symptoms at the time, yet end in death from subsequent hemorrhage. In all cases of sudden death a very accurate knowledge of the woman's previous condition is necessary. Women may suffer from chronic heart disease, aneurism, pancreatitis, etc., which may terminate fatally during or immediately after labor.

---

### PUERPERAL STRANGULATION OF THE SPLEEN.

Prof. Barsany, of Vienna, reports a case in a primipara, aged 26, which showed symptoms of peritonitis owing to torsion of the pedicle of a wandering spleen. On admission she had a high temperature and 17,000 leukocytes, so that a tentative diagnosis of suppurative ovarian cyst was made. Some weeks later, after subsidance of the fever, Barsany opened the abdomen. Fifteen liters of pus were evacuated from an enormous cystic swelling, while near the lumbar vertebræ was found the necrotic spleen with its twisted pedicle. The organ was extirpated and the abscess cavity flushed and drained, after its wall had been sutured to the abdominal wall. It was supposed that the spleen became freely movable after an attack of malaria, and that its pedicle became twisted in the course of the pregnancy, producing hemorrhage into its capsule, which was followed by an infection from the intestine that gave rise to the enormous abscess, although the condition might have been pyemic in its origin.

---

### LABOR OBSTRUCTED BY UNRUPTURED HYMEN.

The following case, which came under my care some time ago in North London, is worth recording. With an experience of over 3,000 labors during the last twenty-three years, this is the only example of the kind I have met, though similar instances are mentioned in obstetrical works. The patient was a young woman who had been married about a year. When summoned to attend, labor had progressed for many hours, for the fetal head was well down on the perineum. The hymen was firm, rigid, and unyielding, and would not admit even the little finger. As this condition was obstructing labor, I made several incisions into the hard, fibrous material, after which delivery proceeded naturally. The perineum was slightly lacerated, but this was purposely left unsutured. The presentation was normal; no complications followed, and the mother subsequently did well.—Edwin Cahill, in *British Med. Jour.*

---

### AN UNUSUAL CASE OF ECTOPIC GESTATION.

CANDLER (*Charlotte Med. Journal*) made a diagnosis of ectopic pregnancy with rupture of the sac into the peritoneal cavity. Owing to the extreme shock from which the patient was suffering the laparotomy was postponed till the next morning. Opiates controlled the pain for a few hours when it returned with all its severity. At 4 A. M. the pains became more violent and cramp-like in character with very little time between them. At this time she acted more like a woman in the last efforts to expel a fetal head through the perineum. There was a sudden cessation of the bearing down pains as if she had succeeded in her task; at the same time there came a gush of liquid, not from the vagina, but from the rectum. What resembled amniotic fluid, shreds of membrane and clots of blood were passed as well. In the course of an hour she again passed about the same quantity of fluid in which was found unmistakable portions of placenta, together with the remains of what was taken for a six weeks fetus. Sanguinous fluid was passed from the rectum for ten days when it ceased and the patient is now enjoying excellent health.

---

### CESAREAN SECTION PERFORMED BY A COW.

W. B. MORSE, Salem, Oregon, (*Journ. A. M. A.*) reports Mrs. W. E., aged 31, pregnant at full term with her third child, and standing in the road about 60 feet from the house, watching her husband unload hay from a wagon. A vicious two-year-old heifer charged her and inserted a very sharp horn into the abdomen near the anterior superiour spinous process of the ilium. It penetrated the abdominal wall, uterus and membranes, but escaped the child. The point of the horn then emerged again just to one side and below the umbilicus. The result was two transverse rents, the upper one about 5 inches long and the other clear across the abdomen. The child was delivered through the larger opening and fell to the road, where it was picked up by the father. I made an eight-mile trip and arrived about 20

minutes after the mother's death from hemorrhage. The uterus was outside the bcdy, torn clear across and inverted, with after-birth and membranes still attached. The child is still alive and doing well. I have found histories of several similar cases in Gould and Pyle's work on medical curiosities.

---

### THE ETIOLOGY OF ECLAMPSIA.

Weichardt and Piltz (*Dcutsch Med Woch.*) say that a study of the mechanism of the production of hay fever and eclampsia leads to the conclusion that each disease is the result of the formation of specific poisons from albumins, in the one case derived from the placental albumin, and in the other from the albumin of the pollen. Weichardt believes that eclampsia is the result of the formation in the body of toxic substances resulting from the cytolisis of placental cells that enter the circulation, it being assumed that in these cases there is a deficiency of anti-toxins or inhibiting bodies. The authors speak of their efforts to produce an artificial inhibitory body to be used as a prophylactic, in which they have been partially successful, though it does not appear that there is any immediate possibility of its being practically useful.

---

### ABORTION FOLLOWING THE USE OF DIACHYLON.

Hope Robeson (*British Medical Journal*) reports the case of a woman who on the advice of a neighbor took pills of diachylon for amenorrhea due to physiological causes. After taking them two weeks she had a miscarriage. Then five months later she had another abortion at two months. The menses had been regular during the second pregnancy and she was not aware that she was pregnant. The typical blue line on the gums was still quite visible.

---

### INJURIES TO THE CHILD'S HEAD DURING LABOR.

Sachs (*Journ. Am. Med. Ass'n.*) warns the obstetrician that, other things being equal and, above all, the life of the mother not being in danger, it is wise to curtail the period of labor as much as possible, and not necessarily to wait until the child's heart action becomes feeble. Many children might have escaped epilepsy, idiocy, and paralysis if the period of labor had been properly managed. He is firmly convinced that protracted labor is the most powerful factor in producing epilepsy, idiocy, or paralysis in the newborn; one or often all of them are developed and may be due to conditions present at the time of birth. He further says that the medical men in attendance at confinements have for years followed a policy of indifference toward the welfare of the child, and have allowed too many children to be born into the world after labor unnecessarily prolonged, and in conditions that are a distinct disadvantage to society and to the individuals for the entire period of their natural lives.

---

### A NEW MANAGEMENT OF OCCIPITO-POSTERIOR POSITIONS OF THE FETAL HEAD.

O'Brien (*Medical Record*) introduces either hand under the occiput, with or without anesthesia, grasps it firmly, having separated the middle and ring fingers to steady the neck, making counter pressure upon the fundus, thus bringing the fetal body and head into one solid mass. With assistance he slowly rotates the mother to the genupectoral posture. This manipulation brings the occiput to the anterior position without traumatism to the mother or child. Labor proceeds as when the patient is placed in the genupectoral position for prolapsed cord, or in the lateral obstetrical posture of the English school. The writer calls attention to the awkward movement which occurs when the patient's knee passes over the accoucheur, for the crux of the manipulation is to keep the head fixed steadily with the body curled upon it.

---

### DRESSING OF WOUNDS.

Miles F. Porter (*American Medicine,* September, 1906) says that unwise efforts to produce asepsis may cause more harm than the bacteria against which they are directed. The surgeon's attitude toward nature should be that of a modest cup-bearer, not that of a usurper. Sutures, redressings, and drains are, at best, necessary surgical evils, hence all sutures should be discarded in favor of the bandage and adhesive plaster when these can be made to serve the same purpose; but when the use of sutures is imperative, then (*a*) buried absorbable sutures should be preferred; buried non-absorbable temporary sutures should be our second choice; through-and-through sutures should be used only on compulsion; and buried permanent sutures discarded except in work on the hollow abdominal viscera. (*b*) Our aim should be to do the first dressing so that it will be the only one needed until healing is accomplished, and when this is impossible our efforts should be directed toward preventing the necessity of frequent dressing. (*c*) When in doubt, do not drain, but if drainage is imperative let it be adequate without being harmful, kept up while it is needed, and discarded so soon as it has fulfilled its mission.—*Medical Age.*

